# Transforming mental health care in a changing Europe

**DOI:** 10.1192/j.eurpsy.2026.12245

**Published:** 2026-07-20

**Authors:** Andrea Fiorillo, Celso Arango

**Affiliations:** 1 Department of Psychiatry, University of Campania Luigi Vanvitelli: Universita degli Studi della Campania Luigi Vanvitelli, Naples, Italy; 2 Hospital Universitario La Paz, IdiPAZ; School of Medicine, Universidad Autónoma de Madrid; CIBERSAM, Madrid, Spain

Europe is undergoing profound social, economic, political, and technological transformation, with direct implications for mental health and the systems designed to support it. As demand rises and pressures intensify, psychiatry is being called to respond at scale, while maintaining quality, equity, and continuity of care. Against this backdrop, the European Psychiatric Association is highly committed to prioritizing mental health in the European agenda, highlighting both the urgency of the challenge and the need for collective action.

The scale of the challenge is clear: recent evidence points to sustained increases in the levels of several mental disorders, including anxiety, depression, psychosis, and sleep disorders, among others, with loneliness and other social determinants contributing to a growing personal and societal burden further increased in the aftermath of the pandemic [1]. Mental disorders account for a significant share of economic cost across EU Member States and the United Kingdom, yet investment in mental health remains below recommended levels, underlining the need for coordinated, system-wide action [2]. The recent increases in the incidence, morbidity, disability, and mortality levels from mental health conditions have not been followed by increased responsiveness by health care sectors and other sectors, creating a “perfect storm” for worsening population mental health [3].

The 2025–2027 Action Plan of the European Psychiatric Association brings together clinical, research, advocacy, and educational priorities aimed at improving quality of care and care delivery across Europe for all those living with mental health conditions, as well as their family members and caregivers [4].

## Mental health care delivery

The effects of these wider social and systemic pressures are not evenly distributed across Europe. Vulnerable groups, including those affected by conflict, displacement, and social marginalization, face heightened risks and greater barriers to care. At the same time, young people are navigating new challenges linked to digital environments, social media use, and uncertainty about the future [5]. These pressures are increasingly shaped by geopolitical instability, forced migration, and widening social inequalities, all of which are placing sustained strain on mental health systems [6, 7].

Meeting these realities demands more than incremental change; it requires mental health systems that are adaptable, evidence-based, and closely connected to the real needs of the communities they serve.

This approach requires rethinking how mental health care is delivered. Expanding mental health care beyond traditional hospital settings, both inpatient and outpatient, into community environments such as schools and workplaces can improve accessibility and reduce stigma, while enabling more flexible, outreaching care delivery and strengthening service delivery models [4, 8].

## Working in collaboration and not in “silos”

Collaboration and partnership are central to the EPA vision and to its leadership role for the future of psychiatry in Europe. This includes close ties with international medical associations, with other relevant medical and brain health societies, regulators and industry partners, as well as with user and carer organizations, such as GAMIAN-Europe and EUFAMI [9, 10]. These partnerships are designed to ensure that a wide range of perspectives, including clinical, educational, scientific, advocacy, and lived experience, are reflected in how priorities are set and implemented across Europe. Rather than being symbolic, they are intended to shape clinical priorities, policy positions, and models of care in a practical and sustainable way. Together, they reflect a broader shift toward co-production in mental health where clinicians, patients, and carers work together to design mental health services that are responsive to real-world needs.

Collaboration also underpins the EPA’s approach to education and leadership. Initiatives such as the EPA Leadership Academy or the European Board Examination of Psychiatry aim to equip early-career psychiatrists with the skills and networks needed to navigate increasingly complex healthcare systems. A sustainable future for psychiatry depends on investment in people as much as in systems.

## From ambition to implementation of the precision psychiatry approach

Innovation must now move from ambition to implementation. Precision psychiatry represents a shift toward more personalized, data-informed care, moving beyond symptom-based diagnosis alone to incorporate clinical history, neurocognitive functioning, physical health, life events, and illness staging. Precision psychiatry follows the experience from other medical disciplines, such as oncology or immunology [11]. Within the EPA Action Plan, this is being translated into more comprehensive assessment frameworks that incorporate neurocognition, biomarkers, determinants of mental health, physical comorbidities, and life course factors [4, 11]. Through the introduction of these assessment frameworks, the EPA supports earlier and more accurate diagnoses, as well as more tailored and personalized treatment pathways, helping to improve patients’ outcomes across Europe.

## A lifespan approach to brain and mental health

Mental health and brain health are closely linked, and both of them must be understood across the lifespan. Many mental health conditions begin in childhood, adolescence, or early adulthood, during periods of significant brain development [12]. Thus, many mental disorders can be prevented with adequate preventive approaches, already in the first years of life, when synaptogenesis and myelination take place [13]. The other crucial phase of development is adolescence, when pruning occurs, and when the prefrontal cortex develops.

Understanding these biological processes is critical for psychiatrists and mental health professionals in general, if we want to deliver effective and tailored early intervention and treatment. Research into brain function, alongside greater integration between psychiatry, neurology, and other medical disciplines, will be essential to improving outcomes across the lifespan.

## Public mental health and prevention of mental disorders

Promoting mental health at a population level is as important as treatment. Although effective evidence-based interventions are available across prevention, treatment, and mental well-being promotion, major implementation gaps persist throughout the European region [8, 13]. Strengthening public mental health through coordinated policy, needs assessment, workforce development, and collaboration with people with lived experience can improve population outcomes, reduce inequalities, and support sustainable social and economic development.

Young people must remain a central focus. With one in seven children and adolescents in the WHO European Region living with a mental health condition, early intervention remains critical [14]. This includes not only clinical support but also sustained efforts to promote mental health literacy and foster environments that support well-being. This is without forgetting the elderly: with the increasing life expectancy, people are increasingly facing life difficulties and disabilities, which may lead to poor mental health [15]. As part of this prevention and promotion agenda, mass media and digital platforms have a key role to play. Responsible, accurate, and stigma-free communication can help shape public understanding, elevate the voices of mental health professionals and experts, and encourage help-seeking behavior.

## Looking ahead: shared responsibility in a changing Europe

These priorities are increasingly shaping the direction of European psychiatry, with a growing focus on translating advances in research into practical improvements in care delivery.

Personalized and integrated care means recognizing the uniqueness of each individual through a “Whole Person Health” approach, while strengthening collaboration across disciplines, services, and sectors. It calls for evidence-based, individualized approaches that are adaptable to real-world clinical practice, supported by innovation, prevention, and early intervention. This is a philosophical and a practical shift, since psychiatrists should be able to look at the person as a whole, not only to his/her illness.

In a changing Europe, the future of mental health care will depend on whether ambition is matched by implementation. This includes strengthening investment in the workforce, expanding access to care, supporting responsible innovation, and ensuring that services are shaped in partnership with the people they serve.

Psychiatry has a central role to play in this transformation. The challenge now is to ensure that advances in science, service design, and policy lead to scalable interventions and measurable improvements in patient outcomes across Europe. By working together across systems and societies and grounding innovation in real-world practice, we can help build a mental health care that is more responsive, more equitable and more resilient.
